# Core–Shell PEDOT-PVDF Nanofiber-Based Ammonia Gas Sensor with Robust Humidity Resistance

**DOI:** 10.3390/bios14090411

**Published:** 2024-08-24

**Authors:** Shenghao Xiao, Mengjie Hu, Yinhui Hong, Mengjia Hu, Tongtong Sun, Dajing Chen

**Affiliations:** School of Pharmacy, Hangzhou Normal University, Hangzhou 311121, China; 2022112025100@stu.hznu.edu.cn (S.X.); 2023112025042@stu.hznu.edu.cn (M.H.); 2023112025039@stu.hznu.edu.cn (Y.H.); 2023112025110@stu.hznu.edu.cn (M.H.); 13796901696@163.com (T.S.)

**Keywords:** ammonia sensors, core–shell, nanofiber, humidity resistance

## Abstract

Current ammonia sensors exhibit cross-sensitivity to water vapor, leading to false alarms. We developed a core–shell nanofiber (CSNF) structure to address these issues, using conductive poly(3,4-ethylenedioxythiophene) (PEDOT) as the core and hydrophobic polyvinylidene fluoride-tetrafluoroethylene (PVDF-TrFE) as the shell. The PEDOT-PVDF CSNF, with a diameter of ~500 nm and a 300 nm thick PVDF layer, showed a superior sensitivity and humidity resistance compared to conventional PEDOT membranes for ammonia concentrations of 10–100 ppm. In humid environments, CSNF sensors outperformed membrane sensors, exhibiting a tenfold increase in performance at 51% relative humidity (RH). This study highlights the potential of CSNF sensors for practical ammonia detection, maintaining a high performance under varying humidity levels.

## 1. Introduction

Gas sensors are in rapidly increasing demand across diverse industries, including emissions monitoring, industrial safety and clinical diagnostics [1,2]. These sensors must maintain a high sensitivity and sustain a dynamic sensing range while operating in environments with various interfering molecules. Conductive polymer-based gas sensors are favored for their ability to operate at room temperature, for their low cost and for their ease of fabrication [3,4,5]. Among these, chemiresistor-type sensors are common, where the conductivity of the polymer changes in response to the analyte gas. However, these polymers often suffer from measurement drift and interference issues [6].

Ammonia, a colorless and flammable gas with a pungent odor, poses significant health risks, with an exposure limit of 25 ppm [7]. In the human body, ammonia results from protein catabolism, and its concentration can indicate liver or kidney disease; usually uremia patients exhale ammonia concentrations in excess of 1.5 ppm. Since most of the sensing mechanisms of gas sensors are due to REDOX reactions, it is difficult to improve the selectivity in a variety of gas environments. Zhao et al. adopted a polyethylpyrrolidone (PVP)-modified ammonia sensor to improve the selectivity of gas sensors [8]. Due to its high-water solubility, contact with ammonia vapors may irritate the eyes, mucous membranes, and respiratory system. Exposure to high concentrations can lead to life-threatening conditions such as pulmonary edema and respiratory failure. Therefore, continuous detection and monitoring of ammonia concentrations is essential for health and safety [9].

Various gas sensors developed to detect low concentrations of ammonia often exhibit a cross-sensitivity to water vapor, posing significant challenges in hazardous gas monitoring applications [10,11,12]. These sensors frequently struggle to distinguish whether resistance changes are caused by ambient humidity or changes in analyte concentration, leading to false alarms and baseline drifts [13]. Therefore, this sensing selectivity issue impedes the integration of novel materials and structures into practical gas monitoring applications. While arrays and algorithms are used to calibrate for humidity, they often fail in highly humid environments [14,15].

Polyvinylidene fluoride (PVDF) is an emerging functional polymer known for its structural flexibility, ease of processing and chemical resistance [16]. Poly(3,4-ethylenedioxythiophene) (PEDOT) has been widely used as a gas-sensing material due to its ability to change conductivity under varying environmental conditions [17,18,19]. Electrospinning PEDOT can produce ultrathin polymeric nanofibers with a promising sensing performance due to their high surface-to-volume ratio [20,21]. However, exposure to water vapor increases device resistance and can degrade polymer, forming insulating patches [22,23]. Conventional coatings can minimize this degradation but sacrifice sensitivity and response time.

To address these challenges, we developed a core–shell nanofiber structure that encapsulates the functional conductive material with a filtration layer, thereby maintaining the sensing properties of the nanomaterial while enhancing its performance in humid environments. The core of the composite fiber is composed of PEDOT, which serves as the functional sensing material. The shell, made of PVDF-tetrafluoroethylene (PVDF-TrFE), was co-electrospun with the conductive core polymer to form core–shell nanofibers (CSNFs). This composite structure not only enhances sensitivity due to the large surface area of the nanofiber but also mitigates humidity influence by encapsulating it with a hydrophobic layer.

In this paper, we reported the development of a core–shell nanofiber structure that effectively addresses sensitivity and humidity interference issues in ammonia gas sensing. Our PEDOT-PVDF CSNF sensor, with a total diameter of approximately 500 nm and a hydrophobic PVDF layer thickness of around 300 nm, exhibited a higher sensitivity and superior humidity resistance compared to conventional PEDOT membranes in the ammonia concentration range of 10 ppm to 100 ppm. This versatile core–shell structure represents a significant advancement of functional material and a practical application of polymer nanostructures in the field of gas sensing.

## 2. Materials and Methods

### 2.1. Materials and Instruments

Poly(3,4-ethylenedioxythiophene) poly(styrenesulfonate) (PEDOT: PSS, 2 wt.% in water, Sigma Aldrich Co. St. Louis, MO, USA). Polyvinylpyrrolidone (PVP, 99.8%, Beijing Huawei Ruike Chemical Co. Beijing, China). PVDF-TrFE powder (70:30, Piezotech, Franch). Ammonia (Jingong Co. Beijing, China, balance gas, dry air, moisture concentration <1 ppm). Dimethylformamide (DMF, 98%), Methyl ethyl ketone (MEK, 99.7%), acetone (≥99.5%), silver paste and all liquid reagents are from Sinopharm Chemical Reagent Co. Shanghai China. Electrospinning needle (Shanghai Hongshuo Electronics Co., Ltd., Shanghai, China). Testing chamber humidity was monitored by Keithley 6517 humidity probe, DC, USA. Core–shell nanofiber was prepared by an electrospinning syringe pump (LSP02-2B LongerPump, Baoding, China) and a DC high voltage power supply (P303 Dongwen High voltage, Tianjin, China). Nanofiber morphology was observed by transmission electron microscopy at 100 keV (TEM, FEI Tecnai F20, Hillsboro, OR, USA) and scanning electron microscopy (SEM, FEI, XL30, Hillsboro, OR, USA). Infrared spectra were detected by a Fourier Transform Infrared Spectrometer (FTIR, Thermo Fisher Scientific Nicolet iS20, Boston, MA, USA). The X-ray photoelectron spectra were measured by an X-ray photoelectron spectrometer (XPS, Thermo Scientific K-Alpha, Boston, MA, USA).

### 2.2. Preparation of CSNF Sensors

A coaxial-needle assembly for core–shell electrospinning was configured so that the inner needle (22 Gauge) was aligned concentrically to the outer needle (14 Gauge). The tip of the inner needle extended slightly beyond the tip of the outer needle. This tip designed for core–shell nanofiber output facilitated the concomitant flow of two solutions, with the shell solution encapsulating the core flow. The core and shell solutions were maintained at flow rates of 0.6 and 0.3 mL/min, respectively, by syringe pumps. A 4 kV voltage was applied on the needle tip to create electrostatic force. To ensure a continuous core flow, 3 wt.% PVP was added in PEDOT to increase solution viscosity. The core solution was prepared by mixing 30 wt.% of PEDOT: PSS with 3 wt.% of PVP in 5 mL DI water. The shell solution contained 12 wt.% of PVDF-TrFE dissolved in 50:50 *v*/*v* DMF and MEK. A rotating drum with patterned copper electrodes was set up as the collection plate 15 cm away from the needle tip, and the picture of the electrospinning setup is shown in Figure S1. The fibers were connected to the electrode by silver paste diluted with acetone. The organic solvent in the silver paste could dissolve the PVDF shell layer and establish electrical connections with the core material. For the control group, the same PEDOT solution with 3 wt.% PVP was spin-coated as a membrane over the patterned electrodes without PVDF coating.

### 2.3. Characterization and Testing of CSNF Sensors

The voltage–current response of the CSNF Sensor was measured by a CHI660 electrochemical workstation at a 3 V DC potential applied across two electrodes. Laboratory air was used as the carrier gas for purging and dilution. Air humidification was achieved via a water-filled bubbler. Ammonia 500 ppm calibration gas was used as the target source, with further dilution in the air manually controlled through a mass flow controller. The PEDOT-PVDF CSNF sensor was initially exposed to ammonia in a dry air environment to determine its response to the pure target gas. The selectivity test was carried out under different humidity conditions to evaluate the CSNF’s capability as a signal transducer in a complex sensing environment. The sensor response was quantified as the relative change of measured resistance, (R − R_0_)/R_0_ × 100% = ΔR/R_0_ (%), where R_0_ is the initial resistance in dry air before exposure, and R is the resistance measured during gas exposure. Sensitivity is defined as the measured electrical current per concentration of ammonia [24].

## 3. Results and Discussion

### 3.1. Design Concept

**Figure 1:**
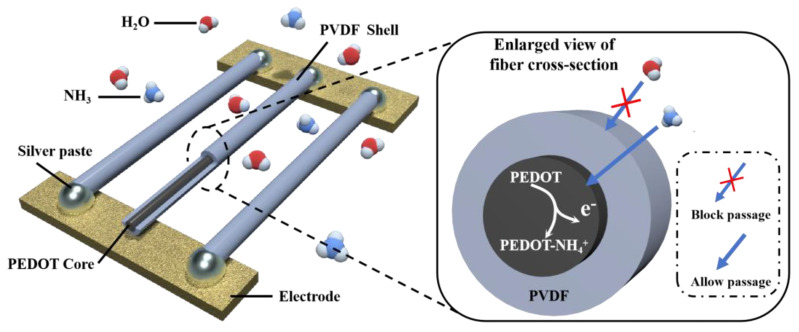
Schematic diagram of the CSNF sensor structure and its response principle.

The successful fabrication of CSNF for gas sensing requires a conductive core capable of efficiently transferring electrical signals to the terminal electrode. PEDOT-based core fibers are particularly promising for such applications due to their superior conductivity compared to Polyaniline and their enhanced stability during the electrospinning process, as PEDOT does not exhibit a granular structure. In order to maintain the sensing performance in humid environments, we proposed a novel core–shell structure design. This design features a conductive PEDOT core as the sensing element and a hydrophobic PVDF shell as the protective layer, as illustrated in Figure 1. The core–shell electrospinning setup consisted of two solution-filled syringes with a concentric needle connected to a high-voltage power supply. The high electric field resulted in substantial charge accumulation that overcame the surface tension of the solution. The syringe pump’s flow rate for the shell component was set higher than the flow rate for the core component, as PVDF solution will easily coagulate in the presence of excess water [25].

### 3.2. Structural and Spectral Characterization of Nanofiber

**Figure 2:**
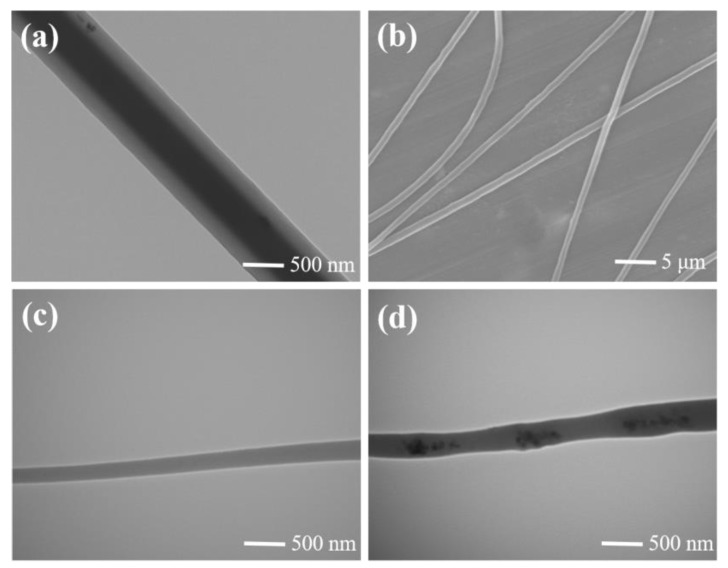
Microscopic images of nanofiber. (**a**) TEM image of PEDOT-PVDF CSNF. (**b**) SEM image of PEDOT-PVDF CSNF. (**c**) TEM image without PEDOT. (**d**) TEM image with PEDOT, but discontinuous.

The nanofiber samples for TEM observation were prepared by directly depositing the coaxially electrospun fibers onto copper grids with a supportive film. The samples were then dried in an oven at 100 °C for 2 h. A typical TEM image of the bi-component nanofiber is shown in Figure 2a. A sharp contrast of the core–shell material along the length of the fiber can be clearly observed due to the differential interaction with the electron beam. This sharp contrast can be attributed to the difference in electron transmission between PEDOT and PVDF. The TEM image also indicated that the composite nanofiber had an overall diameter between 500 nm and 600 nm, with a core diameter below 300 nm. Figure 2b displays the SEM image of PEDOT-PVDF CSNF, uniformly spanning the area between two grounded electrodes. As shown in the SEM image, the diameter of the nanofiber was approximately 600 nm, which aligns with the TEM observation. The rotating drum enabled the formation of an electrospinning nanofiber array with 50 μm gaps. TEM images of PVDF fiber without a core part and PVDF-PEDOT fiber with a discontinuous core are shown in Figure 2c,d.

**Figure 3:**
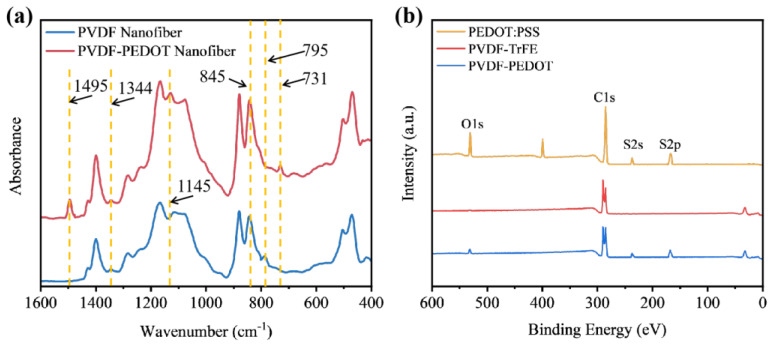
(**a**) FTIR spectra of PEDOT-PVDF CSNF and PVDF nanofibers. (**b**) XPS survey spectra of PEDOT: PSS, PVDF-TrFE and PVDF-PEDOT.

The formation of the core–shell structure is further confirmed by FTIR in Figure 3a. CSNF mats and PVDF nanofiber mats were collected after two minutes of electrospinning. Both the PEDOT-PVDF CSNF and PVDF nanofibers exhibited typical peaks corresponding to the functional groups. The FTIR spectrum of pure PVDF nanofiber mats indicated the co-existence of α-phase and β-phase. The α-phase-related peak was located at 795 cm^−1^, while the β-phase peak, with a higher intensity, appeared at 845 cm^−1^ [26]. The FTIR spectrum of the PEDOT-PVDF core–shell nanofiber showed stretching-vibration absorption peaks of the C=C bond and C-C bond of the thiophene ring in PEDOT at 1495 cm^−1^ and 1344 cm^−1^, respectively. Furthermore, the stretching-vibration absorption peaks of the C-O-C bond were shown at 1145 cm^−1^. Additionally, the C-S bond at 731 cm^−1^ further con-firmed the successful doping of PEDOT: PSS in PVDF [27,28]. To further analyze the chemical compositions of the nanofibers, XPS analysis was carried out on the two pre-pared nanofibers, as shown in Figure 3b. Both nanofibers exhibited characteristic peaks of C and O elements, reflecting their polymer matrices. In addition, S elements (S 2s and S 2p peaks) were only found in PEDOT: PSS and PEDOT-PVDF composites but were not detected in pure PVDF. These results confirmed the compositional differences between the PVDF and PEDOT-PVDF CSNF mats.

### 3.3. Sensing Performance and Selectivity of Gas Sensors

PEDOT is a conjugated polymer with positively charged polarons, which are responsible for its conductivity. When ammonia gas vapor diffuses through the PVDF shell, the PEDOT core interacts with the electrons donated by NH_3_, leading to a redox reaction that significantly increases the sensor’s resistance. This reaction reduces the number of polarons along the PEDOT polymer chain, thereby decreasing its overall conductivity. When a constant voltage was applied across the CSNF, exposure to NH_3_ vapor resulted in a noticeable decrease in current. The hydrophobic nature of the PVDF layer effectively prevented water penetration into the core component.

Due to the highly insulative property of PVDF, the resistance between two electrodes was higher than 1 GΩ. Silver paste was used to connect PEDOT core and electrodes. The organic polar solvents in the silver paste dissolve the PVDF, establishing a conductive pathway to the PEDOT core. Upon covering the core–shell nanofibers with silver paste, the device resistance dropped to 1.5 MΩ, indicating an effective connectivity between the PEDOT core and the electrode. This high conductivity provides PEDOT-PVDF CSNF with a promising electrical property, making it suitable for application as a flexible gas sensor. To evaluate the influence of water condensation on the fiber surface, PVDF fibers without the PEDOT core were electrospun and placed in the same test environment. The fibers were also connected to electrodes using silver paste, and the resistance was measured as higher than 1 GΩ. Compared with the 1.5 MΩ resistance of the PEDOT core, the water condensed on the hydrophobic PVDF surface is not the dominating factor interfering with gas sensing.

**Figure 4:**
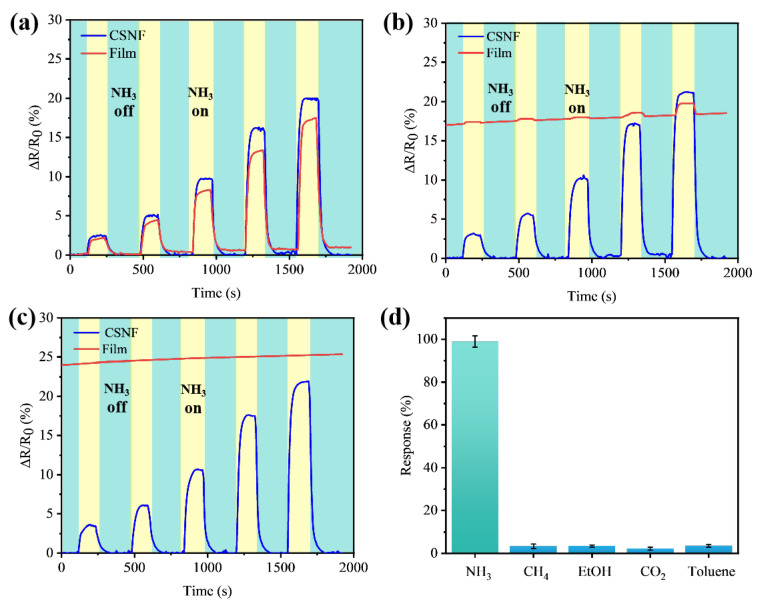
Electrical response of CSNF and PEDOT membranes to different ammonia concentrations (10, 20, 40, 60, 80 ppm) in different humidity environments: (**a**) In a dry environment; (**b**) in a 51% RH environment; (**c**) in a 95% RH environment. (**d**) The CSNF sensor responses to ammonia, methane, ethanol, carbon dioxide and toluene in dry environments (all at 100 ppm).

Figure 4 illustrates the electrical responses of the CSNF and PEDOT membranes to various NH_3_ concentrations (ranging from 10 ppm to 100 ppm) under different humidity environments. In dry air (15% RH), the relative resistance changes of the CSNF and membrane samples exposed to 10 ppm NH_3_ were 2.4% and 1.9%, respectively. Under 51% RH, these changes were 2.9% and 0.3%, respectively. Exposure to water vapor causes polymer swelling, which increases the distance between PEDOT chains and disrupts electron hopping. In the 95% RH environment test, the membrane sensor failed to exhibit an obvious response to NH_3_ due to substantial resistance changes following initial exposure to the humid environment. However, the CSNF sensor demonstrated a 3.2% relative resistance change under 95% RH, which was even higher than its response under 51% RH and 15% RH. This enhanced sensitivity in high humidity is likely due to the high solubility of NH_3_ in water, as NH_3_ dissolves easier in condensed water in a high humidity environment than in dry air.

The response and recovery time are defined as the time required for sensor signals to reach 90% of their stable values. For NH_3_ gas detection, the response time of the membrane sensor was 40 s and the recovery time was less than 80 s. The response and recovery times of the membrane sensor were shorter than those of the CSNF sensor due to the additional coating membrane over the PEDOT core in the CSNF sensor. The response time is influenced by the diffusion of the analyte into the organic sensing layer. Initially, NH_3_ diffuses from the testing environment to the PVDF coating layer interface. NH_3_ then volatilizes through the air–PVDF interface, diffuses across the hundreds-of-nanometers-thick PVDF layer, and finally reaches the PEDOT core, where it conjugates with the sulfur atom of PEDOT. This process continues as gas diffuses into the core, causing an instantaneous reaction that changes the conductivity. Under this configuration, total ammonia diffusion balance is achieved when the ammonia concentration under the PVDF layer equals that of the test environment. The driving force for the process of gas membrane diffusion is the difference in the partial pressure of ammonia between the environment and the core component.

In Figure 4a–c, the resistance of the CSNF sensor almost recovered its initial value after exposure to various concentrations of NH_3_. The minor drift in the current curve over time indicated that the response to NH_3_ was reversible. In contrast, Figure 4b shows that the membrane sensor’s baseline exhibited significant drifting after NH_3_ exposure, due to the swelling effect caused by humidity, which alters the physical properties of PEDOT. Thus, the CSNF structure provided better reproducibility and reversibility. In practical applications, gas sensors are often exposed to complex gas mixtures, making selectivity a crucial parameter. The detection results for different gases at a 100 ppm concentration using the CSNF sensor in a dry environment are shown in Figure 4d. Relative to the response to ammonia, the sensor’s response to methane (CH_4_), ethanol (EtOH), carbon dioxide, and toluene is less than 5% of its response to ammonia.

### 3.4. Resistance to Humidity Interference

**Figure 5:**
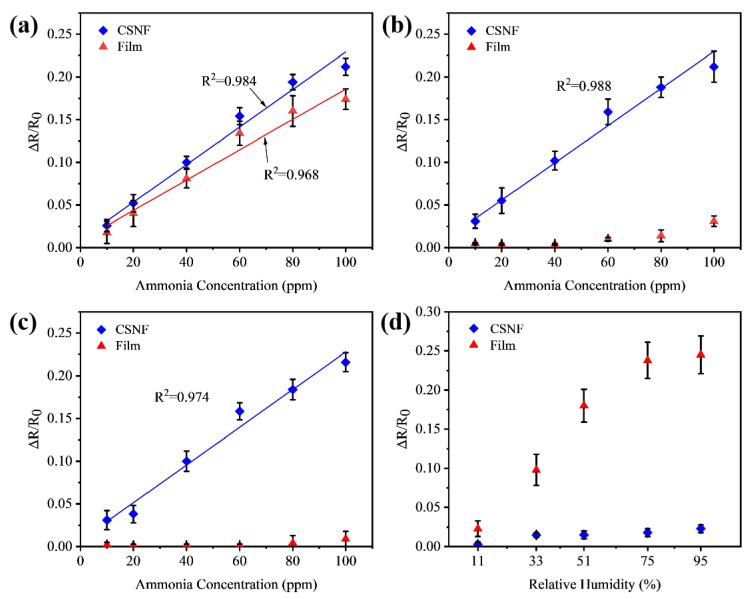
Response of CSNF sensor and PEDOT membrane sensor to ammonia vapor concentration in different humidity environments: (**a**) In a dry environment; (**b**) in a 51% RH environment; (**c**) in a 95% RH environment. (**d**) Comparison of the performance of the CSNF sensor and the PEDOT membrane sensor in response to different humidity levels.

Figure 5 displays the responses of the CSNF sensor and the PEDOT membrane sensor in various humidity environments as a function of NH_3_ vapor concentrations. The resistances of the membrane sensors increased with a rising NH_3_ concentration in the 10–100 ppm range. Notably, the PEDOT-PVDF CSNF sensor exhibited a linear response over this concentration range and demonstrated a higher sensitivity compared to the PEDOT membrane sensor (Figure 5a). In general, gas-sensor sensitivity improves with an increasing surface-area-to-volume ratio. In the case of the CSNF, it is hypothesized that the PVDF coating might reduce the sensitivity because PVDF is a well-known insulating material, which could decrease the effective surface area by coating the PEDOT core. Despite this, the CSNF sensor achieves a similar sensitivity to the membrane structure due to its nanofiber architecture.

The hydrophobic property of the PVDF shell layer prevents condensed water droplets from penetrating the core due to surface tension, resulting in a fixed interface at the shell surface. Since the boiling point of ammonia is much lower than that of water, the concentration difference at the interface produces a vapor pressure gradient. This gradient drives the vapor molecules of more volatile compounds, such as ammonia, to migrate from the droplet to the permeable side of the membrane.

In a humid environment, the core–shell-based device exhibited a superior performance compared to the membrane sensor, as summarized in Figure 5. In low humidity, both sensors showed similar responses to ammonia. However, the linearity coefficient R^2^ of the CSNF sensor in a dry environment was 0.984, which is better than the 0.968 coefficient of the PEDOT membrane sensor. As humidity increased, the performance of the two sensors diverged significantly in terms of ammonia sensitivity. As shown in Figure 5b, the performance of CSNF in a 51% RH environment increased tenfold compared to a conventional membrane device. Figure 5c illustrates that CSNF devices produced even higher signal outputs than membrane devices, which typically lose function in the same concentration range. Figure 5d summarizes the performance comparison of the two sensors under different humidity levels, highlighting that water vapor predominantly affects conductive changes in the unprotected membrane device.

The sensor mainly operates at room temperature. To understand the effect of temperature on sensor performance, we tested the temperature response of the CSNF sensor and the PEDOT membrane sensor at 10–40 °C (20 ppm, 51% RH). The results are shown in Figure S2. Due to the protective effect of PVDF, the drift caused by temperature in the CSNF sensor was lower than that of the PEDOT membrane sensor. In actual testing, temperature calibration can be performed using a temperature sensor. The storage stability of the sensor is shown in Figure S3. The CSNF sensor stored for 28 days was used to detect ammonia with a concentration of 20 ppm. The response on the 28th day was 97.15% of that on the first day, indicating that the CSNF sensor has a high storage stability.

## 4. Conclusions

In this study, a novel ammonia sensor, which utilizes a core–shell nanofiber structure, was fabricated using a coaxial electrostatic spinning method for the detection of ammonia in wide humidity ranges. The conductive polymer PEDOT was used as the sensing layer, while the hydrophobic PVDF served as the protective shell, preventing the infiltration of water vapor and increasing the specific surface area. The experimental results showed that the relative resistance of the CSNF sensor is approximately 10 times higher than that of the conventional PEDOT membrane at 51% RH. The CSNF sensor also showed a high linearity to ammonia in a 95% RH environment. Moreover, the CSNF sensor exhibited an excellent selectivity for ammonia. These results confirmed that PEDOT-PVDF nanofibers are promising sensing materials for ammonia detection in high-humidity environments. The nanostructure developed in this study provided a novel and convenient approach for the advancement of biosensors in various applications, including medical diagnostics, environmental monitoring, and safety control.

## Data Availability

The data that support the findings of this study are available on request from the corresponding author, upon reasonable request.

## Supplementary Materials

The following supporting information can be downloaded at: https://www.mdpi.com/article/10.3390/bios14090411/s1, Figure S1: Physical picture of equipment for preparing core-shell electrospinning. Figure S2: The response of CSNF sensor and the PEDOT membrane sensor to 20 ppm concen-tration of ammonia at 10–40 °C and 51% RH. (b) Storage stability of CSNF sensors in 1–28 days. Figure S3: Storage stability of CSNF sensors in 1–28 days.
