# Supplementary material for: Core–Shell PEDOT-PVDF Nanofiber-Based Ammonia Gas Sensor with Robust Humidity Resistance
Source: Biosensors (Basel). 2024 Aug 24;14(9):411. doi: 10.3390/bios14090411 (PMC11430444; doi:10.3390/bios14090411)
Supplement: Supplementary file 1 [file biosensors-14-00411-s001.zip › biosensors-3124645-supplementary.pdf]

biosensors

MDPI

Supplementary Materials

Core-shell PEDOT-PVDF nanofiber based ammonia gas sensor with robust humidity resistance

Shenghao Xiao [1], Mengjie Hu [1], Yinhui Hong [1], Mengjia Hu [1], Tongtong Sun [1], and Dajing Chen [1]*

[1] School of Pharmacy, Hangzhou Normal University, Hangzhou 311121, China; 2022112025100@stu.hznu.edu.cn (S.X.); 2023112025042@stu.hznu.edu.cn (M.H.); 2023112025039@stu.hznu.edu.cn (Y.H.); 2023112025110@stu.hznu.edu.cn (M.H.); 13796901696@163.com (T.S.);

* Correspondence: djchen@hznu.edu.cn (D.C.)

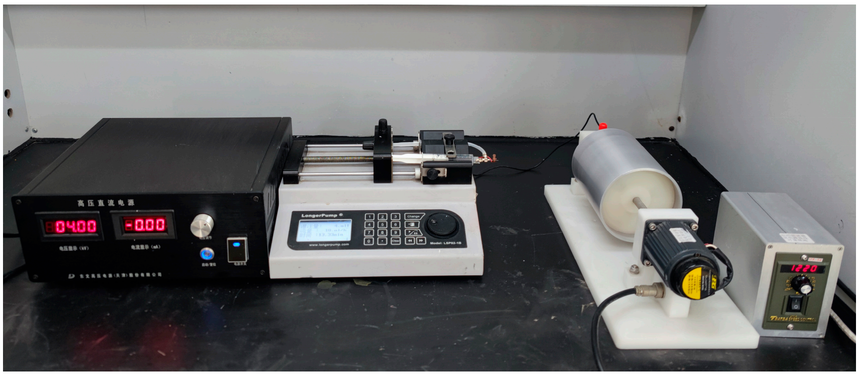

Figure S1. Physical picture of equipment for preparing core-shell electrospinning.

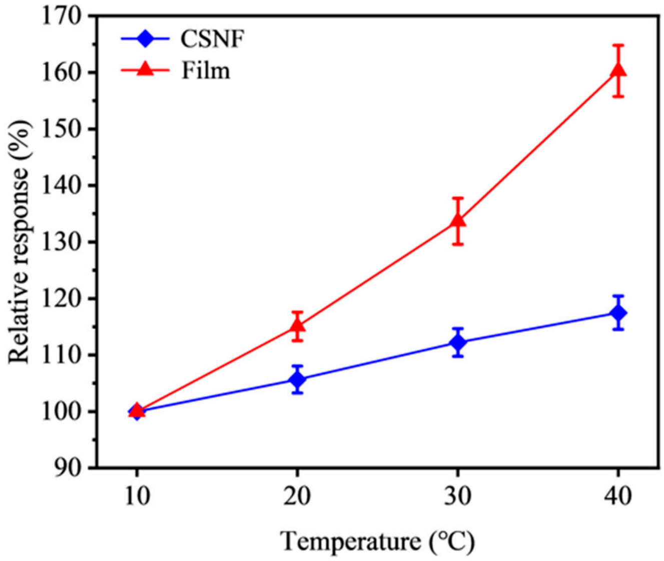

Figure S2. The response of CSNF sensor and the PEDOT membrane sensor to 20 ppm concentration of ammonia at 10-40 ℃ and 51% RH. (b) Storage stability of CSNF sensors in 1-28 days.

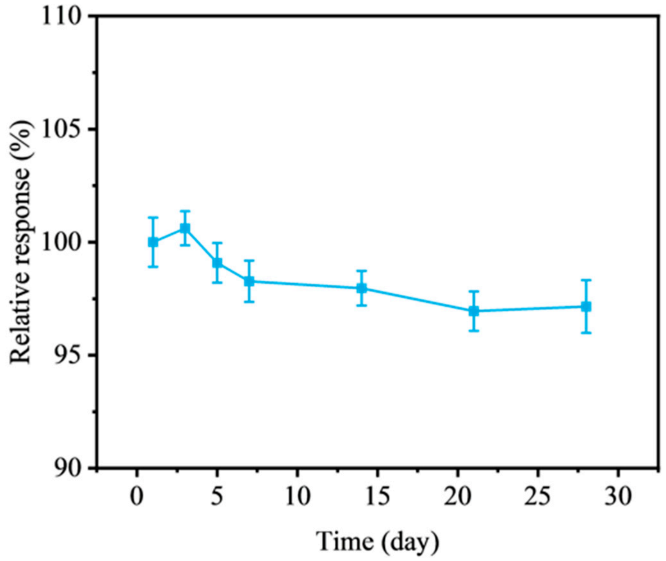

Figure S3. Storage stability of CSNF sensors in 1-28 days.
